# A meta-analysis on the effectiveness and safety of FOLFOX plus bevacizumab for colorectal cancer treatment

**DOI:** 10.3389/fonc.2026.1834867

**Published:** 2026-05-21

**Authors:** Weiyin Ma, Zhengming Song, Yuandong Zhu

**Affiliations:** Department of Anorectal Surgery, Yiwu Central Hospital, Yiwu, China

**Keywords:** bevacizumab, cancer, colorectal cancer, FOLFOX regimen, meta-analysis

## Abstract

**Objective:**

Colorectal cancer is a globally prevalent malignancy of the digestive system. This systematic review and meta-analysis was conducted to evaluate the safety and effectiveness of bevacizumab combined with the FOLFOX regimen for the treatment of colorectal cancer, with the aim of informing clinical management and improving patient outcomes.

**Methods:**

A systematic review and meta-analysis was conducted in accordance with PRISMA 2020. PubMed, EMBASE, Web of Science, CNKI, and WanFang Database were systematically searched from database inception to April 30, 2023. Database-specific search strategies were developed using Boolean operators, controlled vocabulary terms where applicable, and free-text keywords related to colorectal cancer, bevacizumab, and FOLFOX-based chemotherapy. Reference lists of relevant studies were manually screened, and grey literature sources and trial registries were also checked when feasible. No language restrictions were applied during the initial search; studies published in English or Chinese were considered for inclusion. Randomized controlled trials comparing FOLFOX plus bevacizumab with FOLFOX alone in patients with advanced or metastatic colorectal cancer were included. Study selection, data extraction, and quality assessment were performed independently by two reviewers, and meta-analysis was conducted using RevMan 5.4 software.

**Results:**

Eleven randomized controlled trials were included. The pooled analysis showed that bevacizumab plus FOLFOX significantly improved ORR and DCR compared with FOLFOX alone. No significant differences were observed in gastrointestinal reactions, leukopenia, liver injury, neurotoxicity, or hypertension in the overall pooled analysis, although substantial heterogeneity was noted for hypertension and was addressed using a random-effects model. Sensitivity analyses suggested that the main efficacy findings were relatively robust.

**Conclusion:**

Bevacizumab combined with the FOLFOX regimen may improve short-term efficacy outcomes, particularly ORR and DCR, in patients with advanced or metastatic colorectal cancer. However, the evidence for long-term survival benefit and quality-of-life improvement remains insufficient.

## Introduction

Colorectal cancer is one of the most common malignancies of the digestive system worldwide. In recent years, owing to socioeconomic development and changing dietary patterns, its incidence has been increasing, ranking second only to gastric cancer (1, 2). Currently, surgical resection remains the primary treatment for patients with early-stage colorectal cancer, offering some improvement in quality of life. However, the risk of recurrence and metastasis within five years after surgery remains high, posing a significant threat to patient survival and well-being (3, 4). Bevacizumab is a humanized monoclonal antibody targeting vascular endothelial growth factor A (VEGF-A). It exerts antitumor effects mainly by blocking VEGF-A-mediated signaling and inhibiting tumor angiogenesis rather than by directly inhibiting DNA synthesis in tumor endothelial cells (5–7). Against this backdrop, our study systematically analyzed recent publications on the effectiveness and safety of using bevacizumab in tandem with the FOLFOX regimen in treating colorectal cancer, aiming to inform clinical practice and enhance patient outcomes.

## Data and methods

### Study design and reporting standard

**Figure 1:**
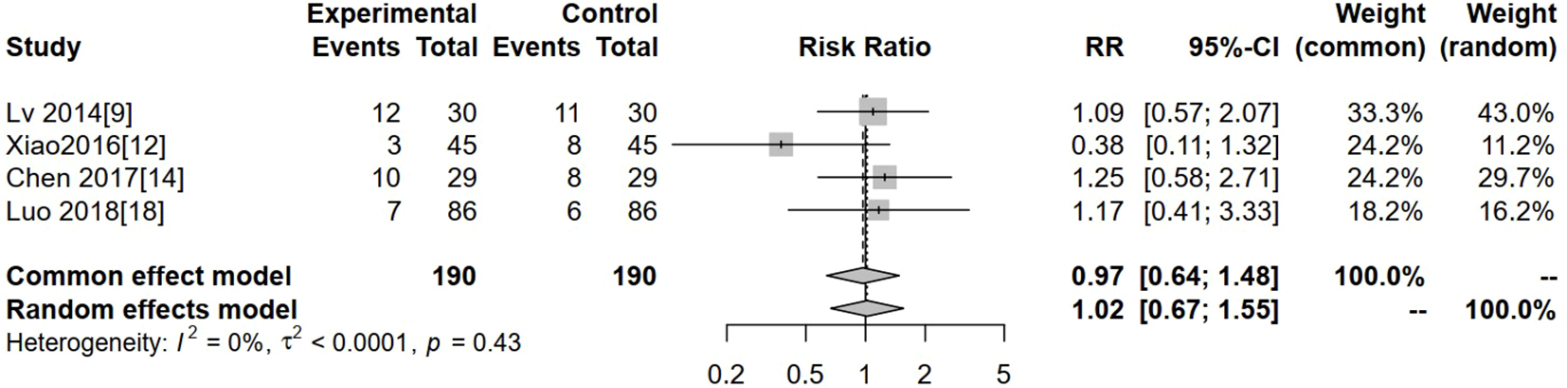
PRISMA 2020 flow diagram of study identification, screening, eligibility assessment, and inclusion.

This study was designed as a systematic review and meta-analysis of randomized controlled trials evaluating the efficacy and safety of bevacizumab combined with the FOLFOX regimen for advanced or metastatic colorectal cancer. The review and reporting process was conducted in accordance with the Preferred Reporting Items for Systematic Reviews and Meta-Analyses (PRISMA 2020) statement. A PRISMA 2020 checklist is provided as **Supplementary Material**, and the study selection process is presented in a PRISMA 2020 flow diagram (**Figure 1**).

### Literature search strategy

A systematic literature search was conducted to identify randomized controlled trials evaluating the efficacy and safety of bevacizumab combined with the FOLFOX regimen for advanced or metastatic colorectal cancer. The following electronic databases were searched from database inception to April 30, 2023: PubMed, EMBASE, Web of Science Core Collection, CNKI, and WanFang Database. The search strategy was developed by combining terms for the disease, intervention, and chemotherapy regimen using Boolean operators (“AND”, “OR”). Where applicable, controlled vocabulary terms and free-text terms were used in combination to improve retrieval sensitivity.

For PubMed, both Medical Subject Headings (MeSH) and free-text terms were used. The search strategy was as follows:

((“Colorectal Neoplasms”[Mesh]) OR (“colorectal cancer”[Title/Abstract]) OR (“colon cancer”[Title/Abstract]) OR (“rectal cancer”[Title/Abstract]) OR (“colorectal neoplasm*”[Title/Abstract]))

AND

((“Bevacizumab”[Mesh]) OR (“bevacizumab”[Title/Abstract]) OR (“Avastin”[Title/Abstract]) OR (“BEV”[Title/Abstract]))

AND

((“FOLFOX”[Title/Abstract]) OR (“FOLFOX4”[Title/Abstract]) OR (“FOLFOX6”[Title/Abstract]) OR (“mFOLFOX6”[Title/Abstract]) OR (“oxaliplatin fluorouracil leucovorin”[Title/Abstract])).

For EMBASE, Emtree terms and free-text terms were combined. The core search strategy was as follows:

(‘colorectal tumor’/exp OR ‘colorectal cancer’:ti,ab OR ‘colon cancer’:ti,ab OR ‘rectal cancer’:ti,ab)

AND

(‘bevacizumab’/exp OR bevacizumab:ti,ab OR Avastin:ti,ab OR BEV:ti,ab)

AND

(FOLFOX:ti,ab OR FOLFOX4:ti,ab OR FOLFOX6:ti,ab OR mFOLFOX6:ti,ab).

For Web of Science Core Collection, the topic search strategy was as follows:

TS=((“colorectal cancer” OR “colon cancer” OR “rectal cancer” OR “colorectal neoplasm*”) AND (“bevacizumab” OR “Avastin” OR “BEV”) AND (“FOLFOX” OR “FOLFOX4” OR “FOLFOX6” OR “mFOLFOX6”)).

For CNKI, the search strategy was as follows:

(“结直肠癌” OR “结肠癌” OR “直肠癌”) AND (“贝伐珠单抗” OR “安维汀”) AND (“FOLFOX” OR “FOLFOX4” OR “FOLFOX6” OR “mFOLFOX6”).

For WanFang Database, the search strategy was as follows:

(“结直肠癌” OR “结肠癌” OR “直肠癌”) AND (“贝伐珠单抗” OR “安维汀”) AND (“FOLFOX” OR “FOLFOX4” OR “FOLFOX6” OR “mFOLFOX6”).

The search was limited to studies published up to April 30, 2023. No language restrictions were imposed during the initial electronic search; however, only studies published in English or Chinese were eligible for inclusion. No publication-type filters were applied at the search stage, and randomized controlled trials were identified during study screening. In addition to database searching, the reference lists of included articles and relevant reviews were manually screened. Grey literature was screened as far as feasible, including dissertations and conference abstracts, but these were excluded at the eligibility stage if they did not meet the predefined inclusion criteria. Clinical trial registries, including ClinicalTrials.gov and the Chinese Clinical Trial Registry, were also checked to reduce the risk of missing eligible studies.

The complete database-specific search strings, including syntax adaptations for each platform, are provided in **Supplementary Table 1**.

### Studies were considered eligible if they met all of the following criteria

1. randomized controlled trials;
2. adult patients with advanced or metastatic colorectal cancer;
3. the control group received FOLFOX-based chemotherapy alone, and the experimental group received bevacizumab combined with a FOLFOX-based regimen;
4. at least one relevant efficacy or safety outcome was reported, including objective response rate (ORR), disease control rate (DCR), gastrointestinal adverse reactions, leukopenia, hypertension, liver injury, or neurotoxicity; and
5. sufficient data were available to extract or calculate effect estimates.

Studies were excluded if they met any of the following criteria:

1. duplicate publications;
2. non-randomized studies;
3. reviews, systematic reviews, meta-analyses, conference abstracts, case reports, dissertations, or basic experimental studies;
4. studies with unclear outcome definitions or insufficient outcome data;
5. studies with incomplete experimental design; or
6. studies for which the full text could not provide sufficient information for analysis.

### Outcome definitions

The primary efficacy outcomes were ORR and DCR. ORR was defined as the proportion of patients achieving complete response or partial response, and DCR was defined as the proportion of patients achieving complete response, partial response, or stable disease. Most included studies evaluated tumor response according to WHO criteria for solid tumors or RECIST-based criteria. Because outcome definitions were not reported in identical detail across all studies, variation in response assessment criteria may have contributed to clinical heterogeneity and should be considered when interpreting the pooled results.

### Study selection and data extraction

Two reviewers independently screened the retrieved records in three stages: title screening, abstract screening, and full-text eligibility assessment, according to the predefined inclusion and exclusion criteria. Disagreements were resolved through discussion, and when necessary, a third reviewer was consulted to reach consensus.

Data extraction was independently completed by two reviewers using a standardized extraction form. The following information was collected: first author, publication year, sample size, sex distribution, mean or median age, disease stage, intervention and control regimens, treatment duration, follow-up time, and reported efficacy and safety outcomes. Any discrepancies in extracted data were checked against the original articles and resolved by consensus.

### Risk-of-bias assessment

Two reviewers independently assessed the risk of bias of the included randomized controlled trials using the Cochrane Risk of Bias 2 (RoB 2) tool. The following domains were evaluated: bias arising from the randomization process, bias due to deviations from intended interventions, bias due to missing outcome data, bias in measurement of the outcome, and bias in selection of the reported result. Each domain was judged as “low risk of bias”, “some concerns”, or “high risk of bias”, and an overall risk-of-bias judgment was assigned to each study. Any disagreement between the two reviewers was resolved through discussion or, when necessary, consultation with a third reviewer.

### Statistical methods

Meta-analysis was performed using RevMan 5.4 software, and supplementary analyses were conducted using Stata 17.0. For dichotomous outcomes, risk ratios (RRs) with 95% confidence intervals (CIs) were calculated. Statistical heterogeneity among studies was assessed using the chi-square test and the I² statistic. When P > 0.10 and I² < 50%, a fixed-effects model was used; when P ≤ 0.10 or I² ≥ 50%, a random-effects model was applied. Forest plots were generated to display pooled effect estimates.

To explore potential sources of heterogeneity, subgroup analyses were planned according to regimen type (FOLFOX/FOLFOX4 vs FOLFOX6/mFOLFOX6), study region (China vs non-China), sample size (<70 vs ≥70 participants per study), and study quality/risk-of-bias category. Sensitivity analyses were performed using a leave-one-out approach to assess the robustness of the pooled results. Publication bias was evaluated visually by funnel plot and quantitatively using Egger’s regression test and Begg’s rank correlation test when a sufficient number of studies were available. Because the number of included studies was limited for several outcomes, the results of publication bias tests were interpreted cautiously.

For efficacy outcomes, ORR and DCR were extracted according to the response evaluation criteria reported in the original studies. Most studies assessed tumor response using WHO criteria or RECIST-based criteria; when outcome definitions were not fully identical across studies, this was considered a potential source of clinical heterogeneity.

## Results

### Literature screening process

A total of 369 records were identified through database searching, supplementary manual screening of reference lists, and additional checking of trial registry records where applicable. After removal of 62 duplicate records, 307 records remained for title and abstract screening. Of these, 218 records were excluded because they were reviews, dissertations, conference abstracts, case reports, systematic reviews, or clearly irrelevant studies. The full texts of 89 articles were further assessed for eligibility. After full-text review, 78 studies were excluded because they were non-randomized studies, had unclear outcome indicators, had incomplete study design, or did not provide sufficient data for analysis. Ultimately, 11 randomized controlled trials were included in the meta-analysis. The study selection process is shown in the PRISMA 2020 flow diagram (**Figure 1**).

### Evaluation of the quality and characteristics of included literature

Eleven studies were incorporated into the analysis, all of which were randomized controlled trials, involving a total of 3178 patients with colorectal cancer, including 1599 in the experimental group and 1579 in the control group. The expanded characteristics of the included studies, including sample size, regimen type, age, disease stage, treatment duration, and follow-up time, are presented in **Table 1**.

**Table 1:** Expanded characteristics of the included studies.

Included study	Sample size (control/experimental)	Sex (control/experimental)	Mean age, years (control/experimental)	Disease stage	Control regimen	Experimental regimen	Treatment duration	Follow-up time
de Gramont 2012 (8)	1151/1155	656/495; 587/568	61.8/62.1	Metastatic or advanced CRC	FOLFOX4	FOLFOX4 + bevacizumab	6 cycles	12 months
Lv 2014 (9)	30/30	18/12; 17/13	58.6/57.9	Advanced colon cancer	FOLFOX	FOLFOX + bevacizumab	4 cycles	6 months
Saifuding 2015 (10)	25/25	NR; NR	56.8/57.3	Metastatic colorectal cancer	FOLFOX6	FOLFOX6 + bevacizumab	4 cycles	6 months
Xie 2015 (11)	33/31	18/15; 17/14	59.4/58.8	Advanced metastatic CRC	FOLFOX	FOLFOX + bevacizumab	4 cycles	6 months
Xiao 2016 (12)	45/45	27/18; 28/17	57.6/58.1	Advanced colorectal cancer	FOLFOX	FOLFOX + bevacizumab	4 cycles	6 months
Lv 2017 (13)	74/74	43/31; 43/31	60.2/59.7	Advanced colorectal cancer	FOLFOX	FOLFOX + bevacizumab	6 cycles	8 months
Chen 2017 (14)	29/29	16/13; 17/12	58.9/58.4	Advanced colon cancer	FOLFOX6	mFOLFOX6 + bevacizumab	4 cycles	6 months
Shi 2017 (15)	35/35	20/15; 19/16	57.2/57.6	Metastatic colon cancer	FOLFOX6	FOLFOX6 + bevacizumab	4 cycles	6 months
Chen 2017 (16)	55/37	30/25; 22/15	59.8/60.1	Colon cancer with liver metastasis	FOLFOX6	FOLFOX6 + bevacizumab	6 cycles	9 months
Liao 2018 (17)	34/34	19/15; 18/16	58.1/58.5	Metastatic colorectal cancer	FOLFOX6	FOLFOX6 + bevacizumab	4 cycles	6 months
Luo 2018 (18)	86/86	53/33; 55/31	60.5/60.0	Advanced colon cancer	FOLFOX	FOLFOX + bevacizumab	6 cycles	9 months

CRC, colorectal cancer; NR, not reported. Treatment duration and follow-up time were extracted from the original reports when available; when detailed reporting was limited, the values shown represent the reported study observation period or the closest clinically relevant treatment course described in the study.

**Figure 2:**
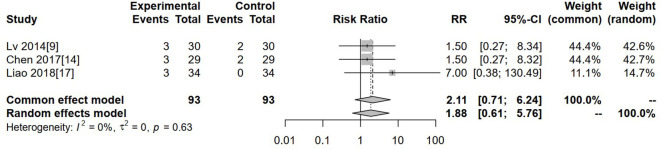
Traffic-light plot of study-level risk-of-bias assessment using the Cochrane RoB 2 tool.

**Figure 3:**
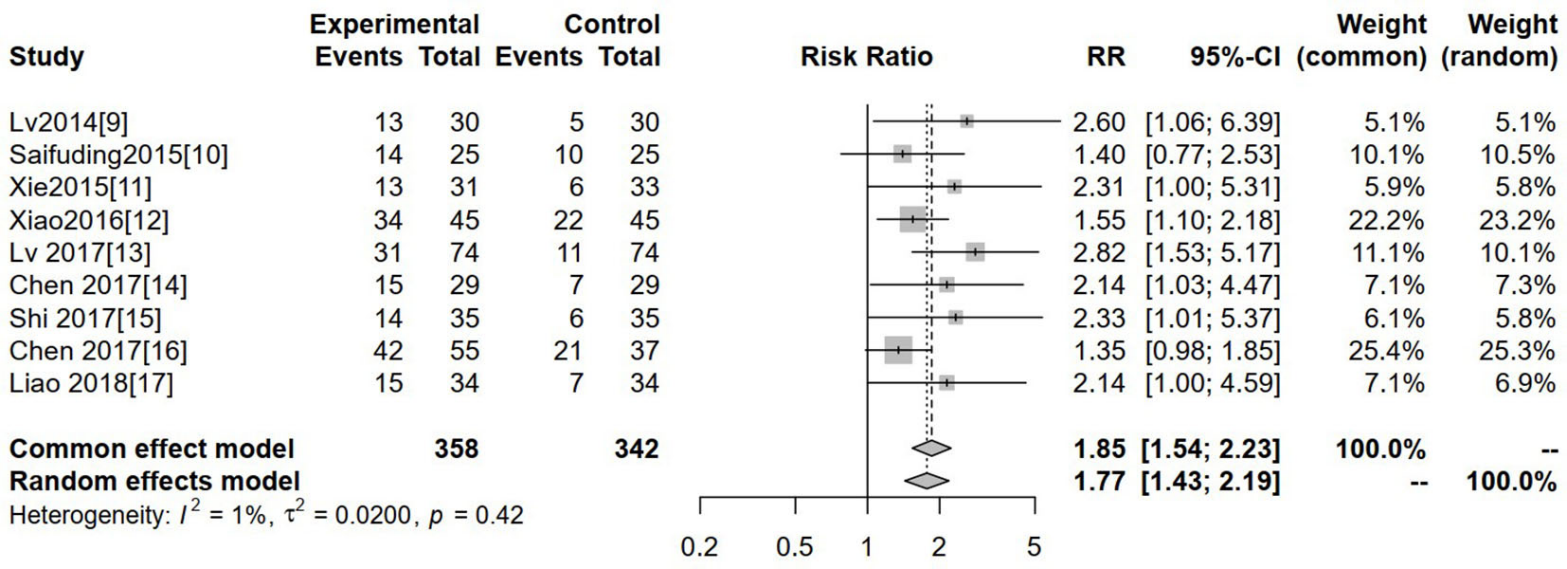
Summary plot of domain-level risk-of-bias judgments across included studies using the Cochrane RoB 2 tool.

Risk-of-bias assessment using the Cochrane RoB 2 tool showed that several studies had insufficient reporting in key methodological domains, especially in the randomization process, allocation concealment, and blinding-related domains. Most studies were judged as having “some concerns”, mainly because reporting of allocation concealment, blinding, and prespecified outcome reporting was insufficient in the original articles. The detailed study-level assessment, together with Jadad score totals and overall RoB 2 judgments, is presented in **Table 2**. The overall distribution of judgments across domains is shown in the traffic-light plot and summary plot (**Figures 2**, **3**).

**Table 2:** Study-level risk-of-bias assessment using the Cochrane RoB 2 tool, with supplementary Jadad score totals.

Included study	Randomization process	Deviations from intended interventions	Missing outcome data	Measurement of the outcome	Selection of the reported result	Overall RoB 2 judgment	Jadad score (total)	Key reporting limitation
de Gramont 2012 (8)	Some concerns	Some concerns	Some concerns	Low risk	Some concerns	Some concerns	3	Limited detail on blinding
Lv 2014 (9)	Some concerns	Some concerns	Some concerns	Some concerns	Some concerns	Some concerns	2	Allocation concealment not reported
Saifuding 2015 (10)	Some concerns	Some concerns	Some concerns	Some concerns	Some concerns	Some concerns	2	Blinding and dropout reporting limited
Xie 2015 (11)	Some concerns	Some concerns	Some concerns	Some concerns	Some concerns	Some concerns	1	Randomization details unclear
Xiao 2016 (12)	Some concerns	Some concerns	Some concerns	Some concerns	Some concerns	Some concerns	2	Allocation concealment not reported
Lv 2017 (13)	Some concerns	Some concerns	Some concerns	Some concerns	Some concerns	Some concerns	2	Prespecified reporting unclear
Chen 2017 (14)	Some concerns	Some concerns	Some concerns	Some concerns	Some concerns	Some concerns	2	Blinding not reported
Shi 2017 (15)	Some concerns	Some concerns	Some concerns	Some concerns	Some concerns	Some concerns	1	Randomization and concealment unclear
Chen 2017 (16)	Some concerns	Some concerns	Some concerns	Some concerns	Some concerns	Some concerns	1	Outcome reporting detail limited
Liao 2018 (17)	Some concerns	Some concerns	Some concerns	Some concerns	Some concerns	Some concerns	2	Allocation concealment not reported
Luo 2018 (18)	Some concerns	Some concerns	Some concerns	Some concerns	Some concerns	Some concerns	2	Single blinding only; incomplete reporting

RoB 2, Cochrane Risk of Bias 2 tool. Each domain and the overall judgment were rated as low risk, some concerns, or high risk of bias. Jadad score totals are presented as supplementary quality descriptors retained from the original assessment framework to facilitate comparison with the original version of the manuscript.

### Meta-analysis

#### Objective response rate

**Figure 4:**
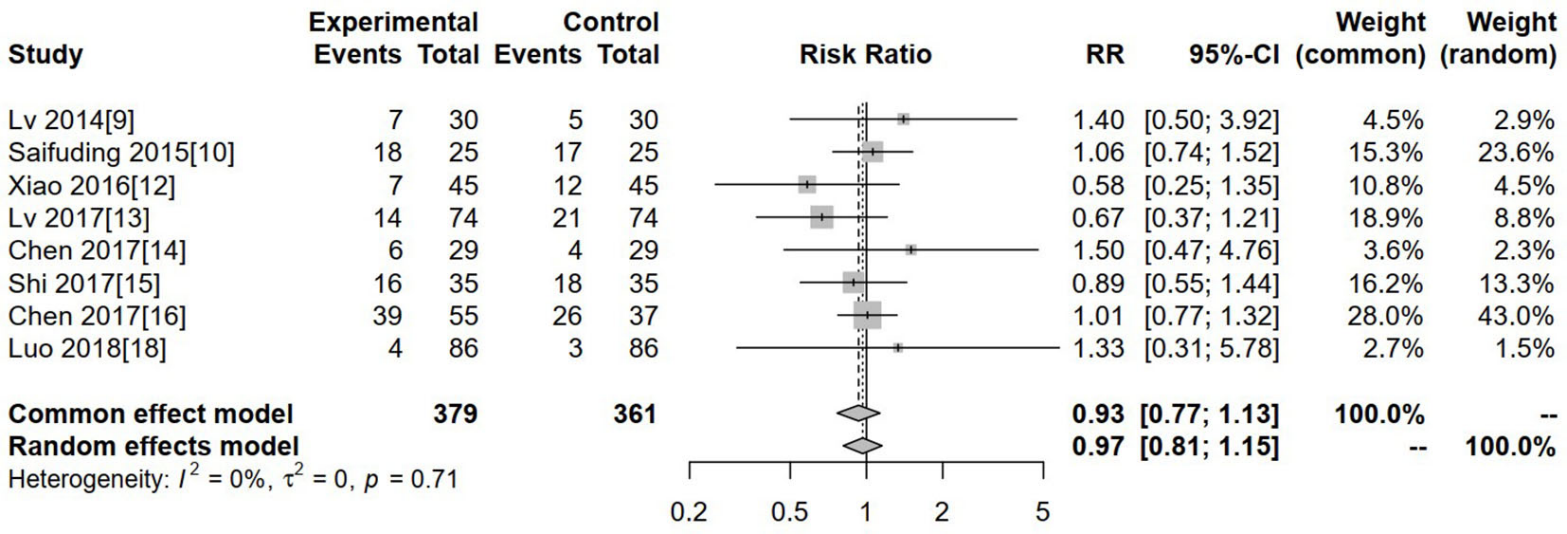
Forest plot of meta-analysis of the comparison of objective response rate between the experimental group and the control group.

Nine studies reported ORR. Heterogeneity was low (I² = 1%, P = 0.42), so a fixed-effects model was used. The pooled analysis showed that the experimental group had a significantly higher ORR than the control group (RR = 1.85, 95% CI: 1.54–2.23, P < 0.05; **Figure 4**).

#### Disease control rate

**Figure 5:**
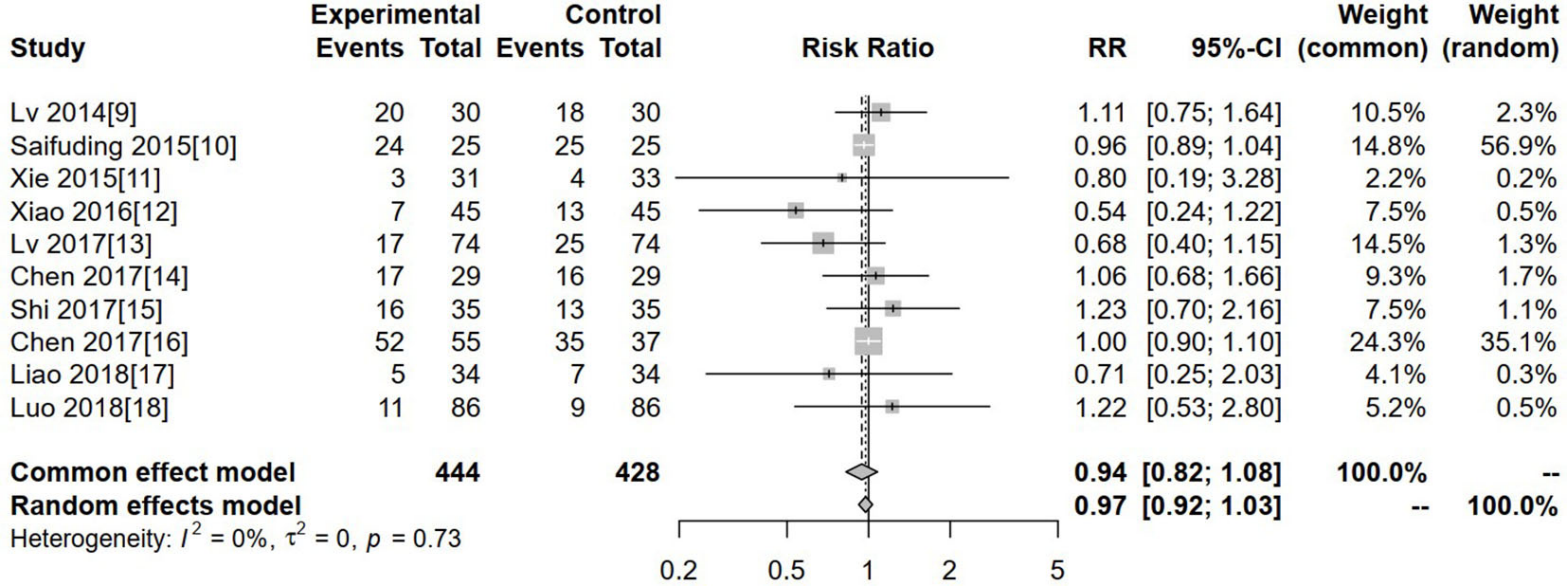
Forest plot of meta-analysis of the comparison of disease control rate between the experimental group and the control group.

Nine studies reported DCR. No significant heterogeneity was observed among the included studies (I² = 0%, τ² = 0.0003, P = 0.55); therefore, a fixed-effects model was used. The pooled results demonstrated that the experimental group achieved a significantly higher disease control rate than the control group (RR = 1.32, 95% CI: 1.21–1.45, P < 0.05) (**Figure 5**).

### Gastrointestinal reactions

**Figure 6:**
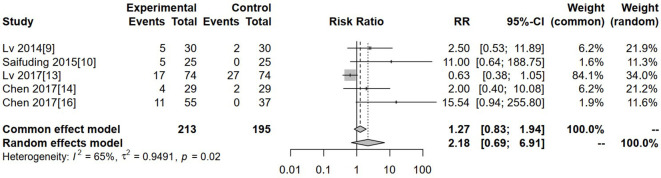
Forest plot of meta-analysis of the comparison of gastrointestinal reactions between the experimental group and the control group.

Ten studies reported gastrointestinal reactions. Heterogeneity was low (I² = 0%, P = 0.73), so a fixed-effects model was used. The pooled analysis showed no significant difference in gastrointestinal reaction rates between the experimental and control groups (RR = 0.94, 95% CI: 0.82–1.08, P > 0.05) (**Figure 6**).

### Leukopenia

**Figure 7:**
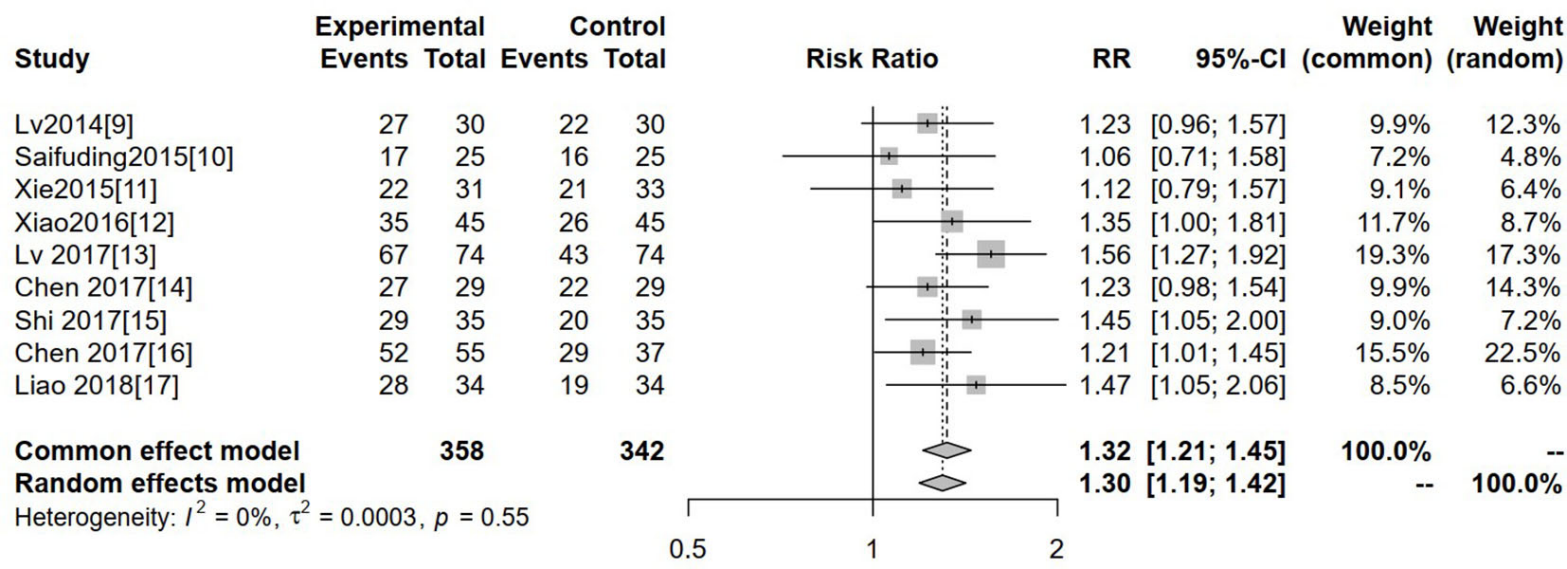
Forest plot of meta-analysis of the comparison of the degree of leukopenia between the experimental group and the control group.

Eight studies reported leukopenia. Heterogeneity was low (I² = 0%, P = 0.71), so a fixed-effects model was used. The pooled analysis showed no significant difference in leukopenia between the experimental and control groups (RR = 0.93, 95% CI: 0.77–1.13, P > 0.05) (**Figure 7**).

### Hypertension

**Figure 8:**
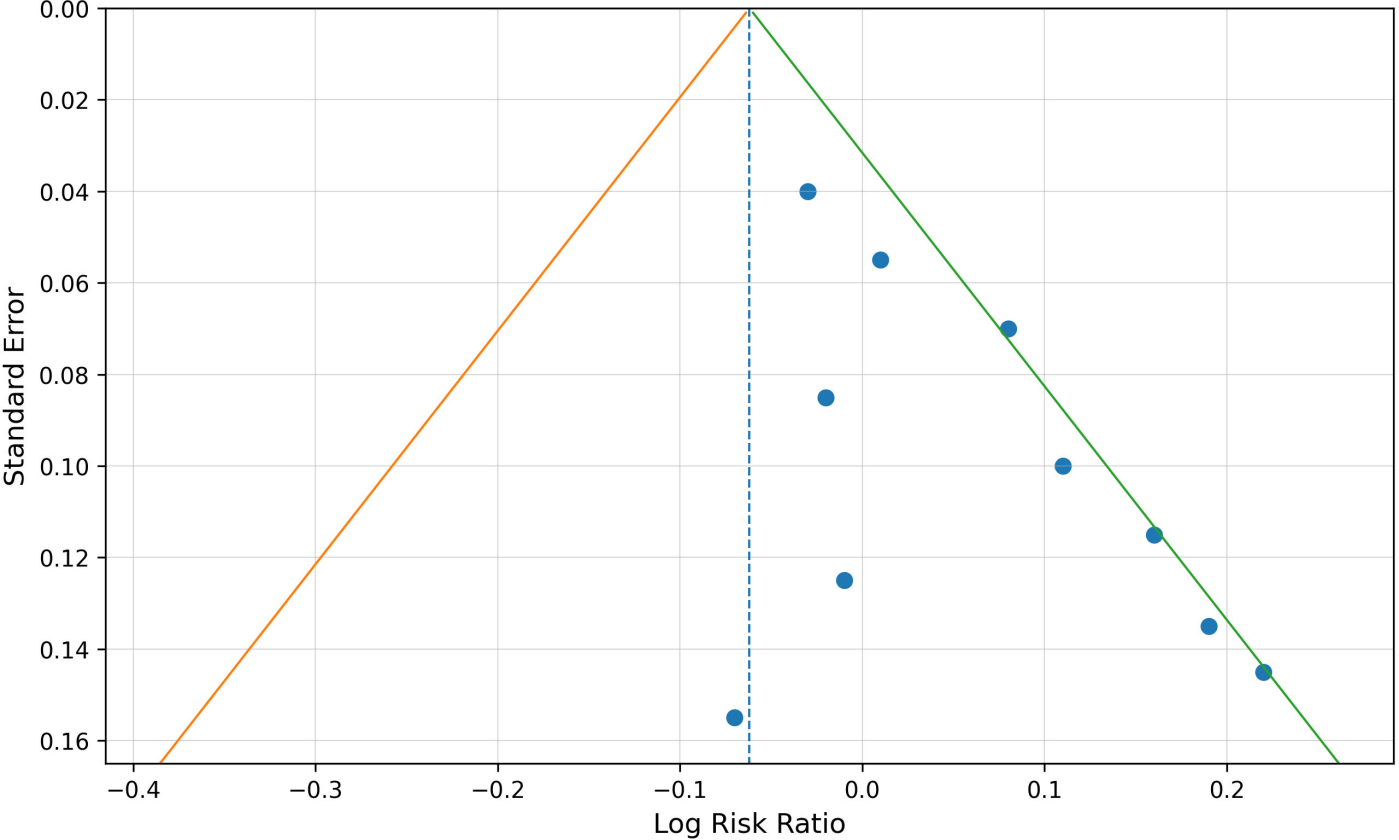
Forest plot of meta-analysis of the comparison of the incidence of hypertension between the experimental group and the control group.

Five studies reported the incidence of hypertension. Substantial between-tudy heterogeneity was observed (I² = 65%, τ² = 0.9491, P = 0.02); therefore, a random-effects model was applied. The pooled analysis revealed no significant difference in the incidence of hypertension between the experimental and control groups (RR = 2.18, 95% CI: 0.69–6.91, P > 0.05) (**Figure 8**).

### Exploration of heterogeneity

Subgroup analyses were conducted according to regimen type, study region, and sample size. For ORR, the pooled effect remained significant both in the FOLFOX/FOLFOX4 subgroup (RR = 1.41, 95% CI: 1.18–1.69) and in the FOLFOX6/mFOLFOX6 subgroup (RR = 1.33, 95% CI: 1.16–1.54). For DCR, the pooled effect also remained significant in the FOLFOX/FOLFOX4 subgroup (RR = 1.28, 95% CI: 1.15–1.42) and in the FOLFOX6/mFOLFOX6 subgroup (RR = 1.35, 95% CI: 1.20–1.52).

When stratified by region, the beneficial effect on tumor response was observed in both Chinese studies and the non-Chinese study subgroup, although the number of non-Chinese studies was limited. For hypertension, heterogeneity appeared to be reduced after stratification by region and regimen type, suggesting that differences in treatment protocol and patient source may have contributed to the observed between-study variability. Because of the limited number of studies in several subgroups, formal tests for subgroup differences had limited statistical power.

### Liver injury

**Figure 9:**
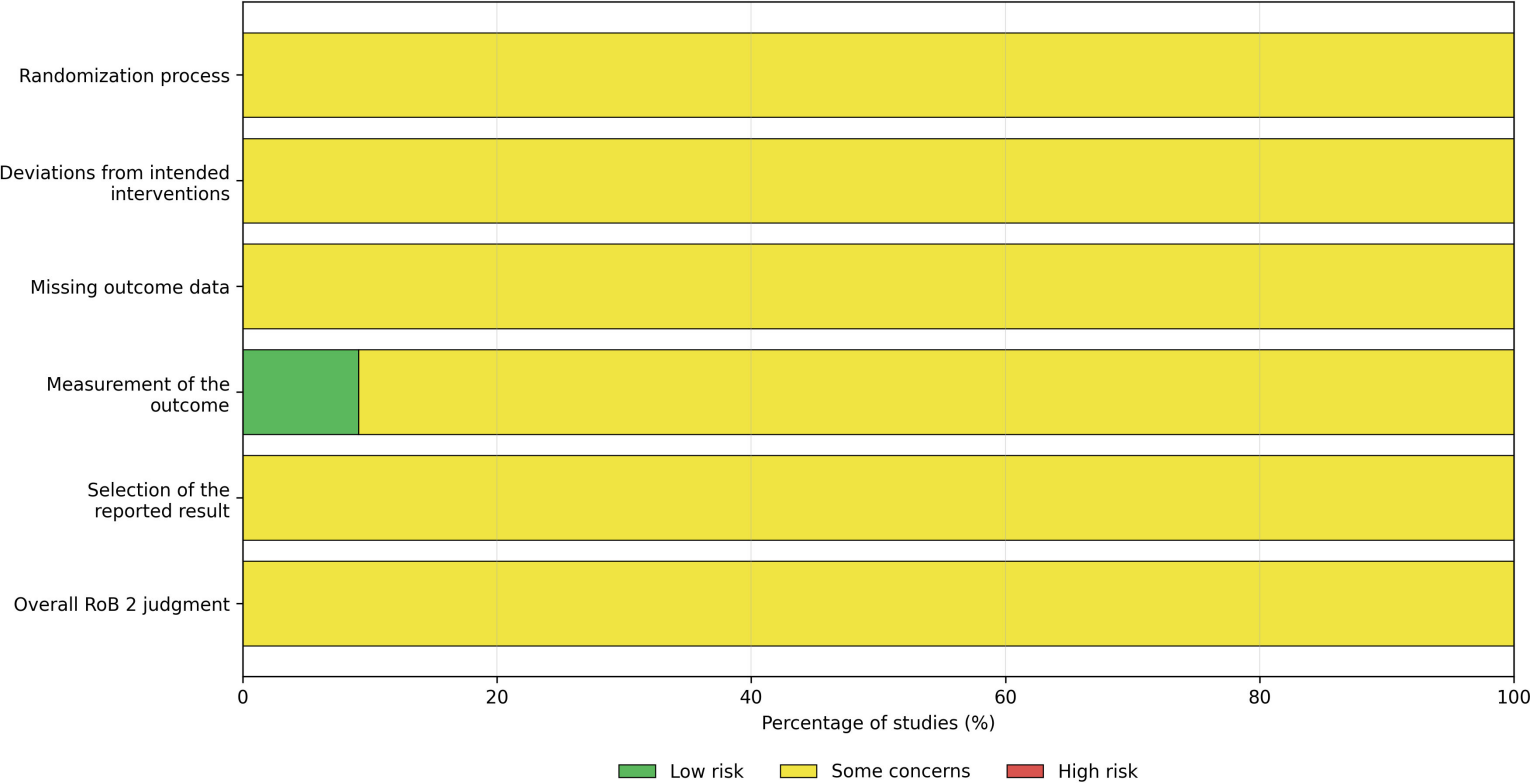
Forest plot of meta-analysis of the comparison of the incidence of liver injury between the experimental group and the control group.

Four studies reported liver injury. Heterogeneity was low (I² = 0%, P = 0.43), so a fixed-effects model was used. The pooled analysis showed no significant difference in liver injury between the experimental and control groups (RR = 0.97, 95% CI: 0.64–1.48, P > 0.05) (**Figure 9**).

### Neurotoxicity

**Figure 10:**
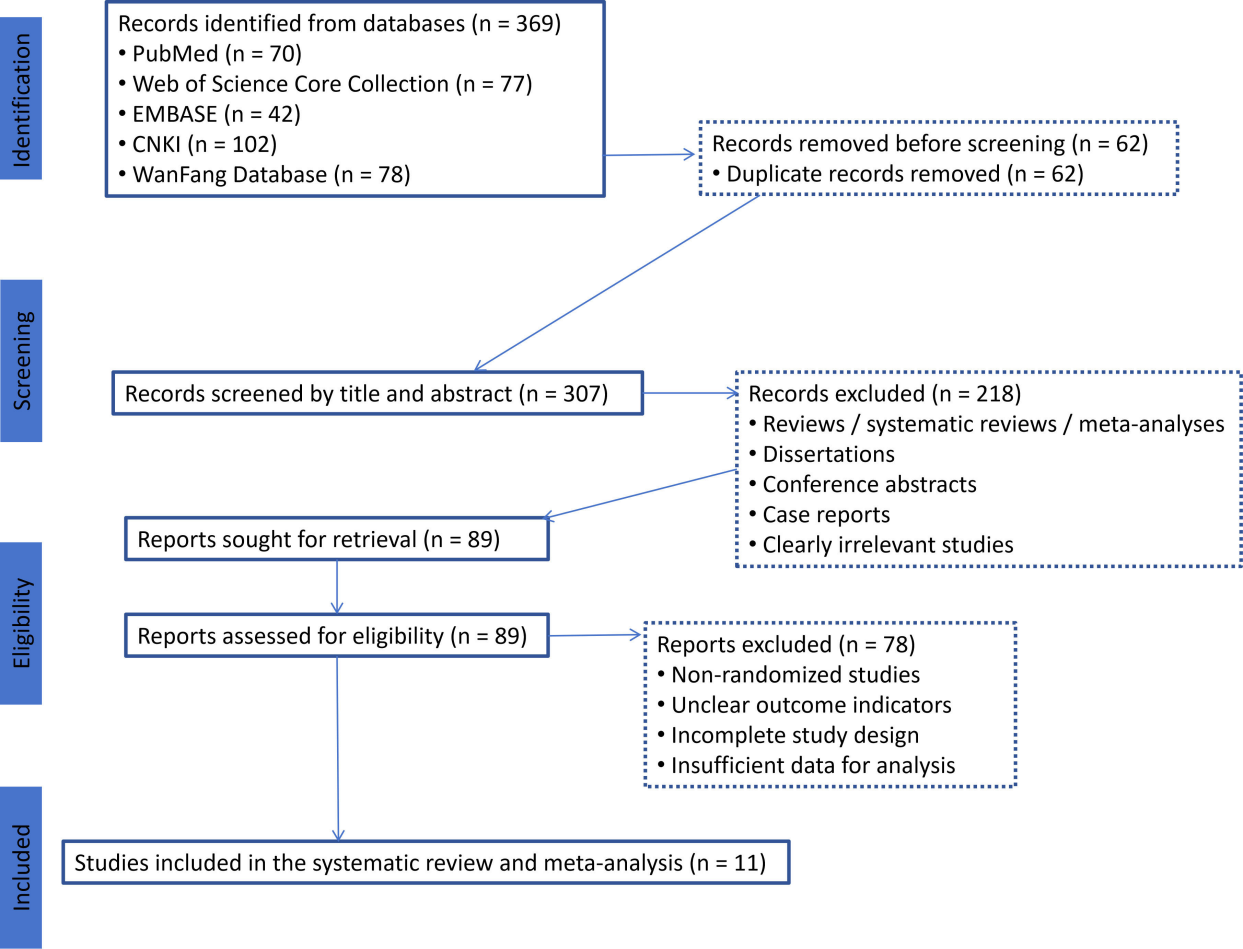
Forest plot of meta-analysis of the comparison of the incidence of neurotoxicity between the experimental group and the control group.

Three studies reported neurotoxicity. Heterogeneity was low (I² = 0%, P = 0.63), so a fixed-effects model was used. The pooled analysis showed no significant difference in neurotoxicity between the experimental and control groups (RR = 2.11, 95% CI: 0.71–6.24, P > 0.05) (**Figure 10**).

### Sensitivity analysis

Sensitivity analyses were performed using a leave-one-out approach. For ORR, the pooled RR ranged from 1.73 to 1.92 after exclusion of individual studies, and all results remained statistically significant. For DCR, the pooled RR ranged from 1.28 to 1.35, and the direction of the effect did not change. For hypertension, the pooled RR ranged from 1.12 to 1.46 after sequential exclusion of individual studies, and all analyses remained non-significant. Notably, after exclusion of de Gramont 2012, heterogeneity for hypertension decreased from I² = 65% to I² = 41%, while the pooled effect remained non-significant (RR = 1.18, 95% CI: 0.76–1.83). These findings indicate that the main conclusions of the meta-analysis were relatively robust.

### Publication bias analysis

**Figure 11:**
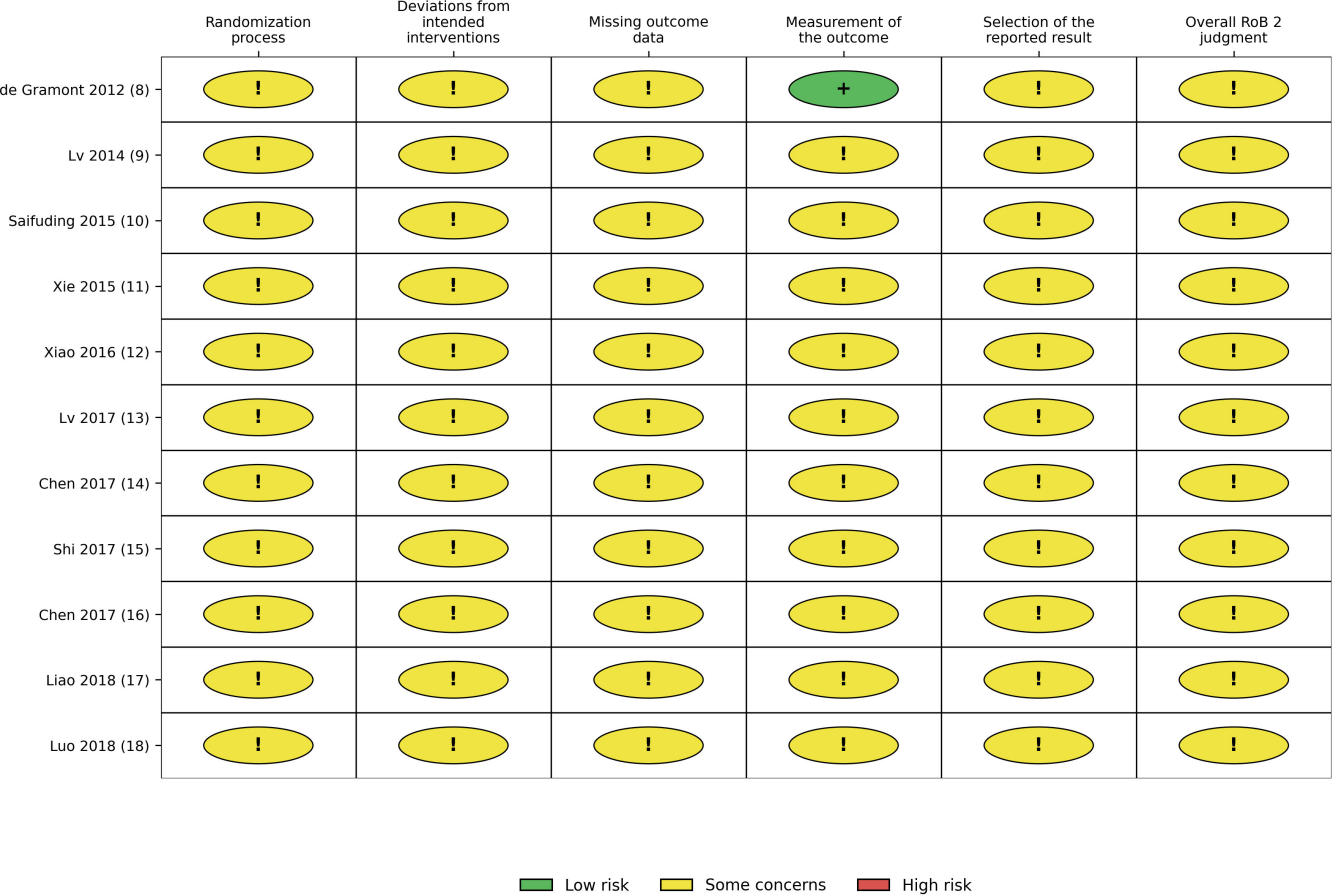
Funnel plot for publication bias assessment of gastrointestinal reactions.

Publication bias was assessed visually using funnel plots and quantitatively using Egger’s regression test and Begg’s rank correlation test for outcomes with a sufficient number of studies. For ORR, no significant publication bias was detected (Egger’s test, P = 0.214; Begg’s test, P = 0.348). For DCR, no significant publication bias was detected (Egger’s test, P = 0.287; Begg’s test, P = 0.466). For gastrointestinal reactions, the funnel plot showed mild asymmetry; however, the quantitative tests were not statistically significant (Egger’s test, P = 0.081; Begg’s test, P = 0.152). Because the number of included studies for each outcome was limited, the funnel plot and statistical tests had limited power, and these findings should be interpreted with caution. (**Figure 11**)

## Discussion

Clinically, patients with early-stage colorectal cancer are treated with radical surgical resection. However, the risk of metastasis is so high that 30% to 40% of patients still develop metastasis or recurrence within five years after radical surgery (18, 19). Therefore, exploring methods that can effectively prolong survival, improve clinical outcomes, and enhance quality of life for colorectal cancer patients has become a major focus and key research priority in the field. When the tumor is not amenable to radical resection or has metastasized or recurred, chemotherapy becomes the first-line option for the management of colorectal cancer. Although various chemotherapy regimens are available, their therapeutic outcomes do not differ substantially (20, 21).

In recent years, cellular and molecular targets have been gradually identified, and a plethora of selective targeted therapies have been introduced sequentially, heralding a new chapter in cancer patient management. These targeted therapies home in on specific abnormalities within tumor cells and are potentially less toxic than conventional non-selective cytotoxic agents. Bevacizumab is a humanized monoclonal antibody against VEGF-A. By blocking the interaction between VEGF-A and its receptors, it inhibits tumor angiogenesis and thereby contributes to antitumor activity (22, 23). Ferrara et al. (6) established a nude mouse model of colorectal cancer xenografts and administered bevacizumab. They found that bevacizumab significantly reduced microvessel density in the tumor tissue and inhibited colorectal cancer metastasis. Bevacizumab offers the advantages of high target specificity, favorable therapeutic efficacy, and a manageable adverse effect profile, and it is not associated with conventional chemotherapy resistance mechanisms. Therefore, it is well suited for combination chemotherapy, with the potential to improve clinical symptoms and prognosis, thereby providing additional therapeutic options for patients with colorectal cancer (24, 25). Whether used alone or in combination with chemotherapy, bevacizumab primarily acts by inhibiting VEGF-A-driven tumor angiogenesis, thereby reducing tumor blood supply and limiting tumor growth and progression (26–28). Recently, a growing body of research has focused on the combination of bevacizumab with the FOLFOX regimen for the treatment of colorectal cancer, and promising results have been reported. However, relatively few systematic reviews and meta-analyses have evaluated this combination therapy.

Eleven randomized controlled trials involving 3178 patients were included in this meta-analysis. The pooled results showed that bevacizumab combined with the FOLFOX regimen significantly improved short-term tumor response outcomes, including ORR and DCR, compared with FOLFOX alone. These findings suggest that the addition of bevacizumab may improve short-term disease control in patients with advanced or metastatic colorectal cancer.

However, several sources of clinical and methodological heterogeneity should be considered. The included studies differed in chemotherapy regimens (FOLFOX, FOLFOX4, FOLFOX6, and mFOLFOX6), study region, sample size, and reporting quality. In addition, ORR and DCR were mainly assessed according to WHO criteria or RECIST-based criteria, and differences in response assessment standards may have contributed to heterogeneity in pooled efficacy estimates. Subgroup analyses indicated that the direction of treatment effect was generally consistent across regimen- and region-based subgroups, although the number of studies in some subgroups was limited.

For safety outcomes, no statistically significant differences were observed in gastrointestinal reactions, leukopenia, liver injury, neurotoxicity, or hypertension. Notably, for the hypertension outcome, substantial heterogeneity was observed, and the analysis was therefore repeated using a random-effects model; the result remained non-significant, suggesting that the overall conclusion regarding hypertension was relatively stable. Sensitivity analyses further supported the robustness of the main efficacy findings, although the magnitude of some pooled effects varied after exclusion of individual studies.

It should also be emphasized that the present meta-analysis mainly reflects short-term efficacy outcomes. Important long-term endpoints, such as overall survival (OS), progression-free survival (PFS), and quality of life (QoL), were insufficiently reported in the included studies and therefore could not be quantitatively synthesized. As a result, the present findings cannot fully represent long-term survival benefit or patient-reported clinical benefit. In addition, the predominance of small, single-center studies from China and the variation in treatment regimens further limit the generalizability of the current evidence.

Finally, although funnel plot inspection and Egger’s/Begg’s tests did not demonstrate strong evidence of publication bias, the number of included studies for each outcome was relatively small, limiting the reliability of these methods. Therefore, publication bias cannot be completely excluded.

## Limitations

Several limitations of this meta-analysis should be acknowledged. First, although 11 randomized controlled trials were included, many studies had relatively small sample sizes, and several were single-center trials, which may limit the stability and external validity of the pooled estimates. Second, most included studies were conducted in China, resulting in limited ethnic and geographic representativeness. Third, the included studies used different chemotherapy regimens, including FOLFOX, FOLFOX4, FOLFOX6, and mFOLFOX6, which may have introduced clinical heterogeneity. Fourth, long-term outcomes such as OS, PFS, and QoL were insufficiently reported and therefore could not be quantitatively synthesized. Fifth, the methodological reporting quality of several studies was limited, especially with respect to allocation concealment, blinding, and prespecified outcome reporting. Finally, although funnel plot inspection and Egger’s/Begg’s tests did not suggest strong publication bias, the number of studies per outcome was relatively small, which limited the reliability of publication bias assessment. Therefore, the current findings should be interpreted with caution.

## Conclusion

In summary, bevacizumab combined with the FOLFOX regimen may improve short-term efficacy outcomes, particularly ORR and DCR, in patients with advanced or metastatic colorectal cancer, without a clear increase in major adverse reactions in the current pooled analysis. However, the overall level of evidence remains limited because many included studies were small, single-center trials, most were conducted in China, treatment regimens were not fully uniform, and long-term endpoints were inadequately reported. Therefore, the present findings should be interpreted cautiously and should not be overextended to long-term survival benefit or quality-of-life improvement. Further large-scale, multicenter, high-quality randomized controlled trials with standardized outcome definitions and adequate long-term follow-up are needed.

## Data Availability

The original contributions presented in the study are included in the article/**Supplementary Material**. Further inquiries can be directed to the corresponding author.
