# Supplementary material for: A meta-analysis on the effectiveness and safety of FOLFOX plus bevacizumab for colorectal cancer treatment
Source: Front Oncol. 2026 May 21;16:1834867. doi: 10.3389/fonc.2026.1834867 (PMC13233368; doi:10.3389/fonc.2026.1834867)
Supplement: Supplementary file 1 [file Table1.docx]

**Supplementary Table S1. Full database-specific search strategies**

This table summarizes the reproducible search strategies used for study identification. The previously reported term "Bedarzab" was corrected to "bevacizumab" for all databases. Searches covered records from database inception to April 30, 2023.

| **Database / source** | **Platform-specific search strategy** | **Controlled vocabulary / subject headings** | **Free-text terms and Boolean operators** | **Limits / filters / notes** |
| --- | --- | --- | --- | --- |
| PubMed | (("Colorectal Neoplasms"[Mesh]) OR ("colorectal cancer"[Title/Abstract]) OR ("colon cancer"[Title/Abstract]) OR ("rectal cancer"[Title/Abstract]) OR ("colorectal neoplasm*"[Title/Abstract]))  AND  (("Bevacizumab"[Mesh]) OR ("bevacizumab"[Title/Abstract]) OR ("Avastin"[Title/Abstract]) OR ("BEV"[Title/Abstract]))  AND  (("FOLFOX"[Title/Abstract]) OR ("FOLFOX4"[Title/Abstract]) OR ("FOLFOX6"[Title/Abstract]) OR ("mFOLFOX6"[Title/Abstract]) OR ("oxaliplatin fluorouracil leucovorin"[Title/Abstract])) | MeSH: "Colorectal Neoplasms"; "Bevacizumab". | OR within disease/intervention/regimen blocks; AND across blocks. | Coverage: inception to 2023-04-30. No publication-type filter at search stage. No language restriction in initial retrieval; English and Chinese studies were eligible at screening. |
| EMBASE | ('colorectal tumor'/exp OR 'colorectal cancer':ti,ab OR 'colon cancer':ti,ab OR 'rectal cancer':ti,ab OR 'colorectal neoplasm*':ti,ab)  AND  ('bevacizumab'/exp OR bevacizumab:ti,ab OR Avastin:ti,ab OR BEV:ti,ab)  AND  (FOLFOX:ti,ab OR FOLFOX4:ti,ab OR FOLFOX6:ti,ab OR mFOLFOX6:ti,ab OR 'oxaliplatin fluorouracil leucovorin':ti,ab) | Emtree: 'colorectal tumor'/exp; 'bevacizumab'/exp. | Title/abstract free-text synonyms; OR within blocks; AND across blocks. | Coverage: inception to 2023-04-30. No study-design filter during searching; RCTs were selected during screening. |
| Web of Science Core Collection | TS=(("colorectal cancer" OR "colon cancer" OR "rectal cancer" OR "colorectal neoplasm*") AND ("bevacizumab" OR "Avastin" OR "BEV") AND ("FOLFOX" OR "FOLFOX4" OR "FOLFOX6" OR "mFOLFOX6" OR "oxaliplatin fluorouracil leucovorin")) | No controlled vocabulary; topic-field search used. | TS included title, abstract, author keywords, and Keywords Plus. OR within blocks; AND across blocks. | Coverage: inception to 2023-04-30. No document-type filter at search stage. |
| CNKI | ("结直肠癌" OR "结肠癌" OR "直肠癌")  AND  ("贝伐珠单抗" OR "安维汀")  AND  ("FOLFOX" OR "FOLFOX4" OR "FOLFOX6" OR "mFOLFOX6") | Chinese subject terms and keywords used according to database indexing and keyword matching functions. | Chinese free-text synonyms combined with OR within blocks and AND across blocks. | Coverage: inception to 2023-04-30. CNKI records were screened for eligibility in Chinese. |
| WanFang Database | ("结直肠癌" OR "结肠癌" OR "直肠癌")  AND  ("贝伐珠单抗" OR "安维汀")  AND  ("FOLFOX" OR "FOLFOX4" OR "FOLFOX6" OR "mFOLFOX6") | Chinese subject terms and keywords used according to database indexing and keyword matching functions. | Chinese free-text synonyms combined with OR within blocks and AND across blocks. | Coverage: inception to 2023-04-30. WanFang records were screened for eligibility in Chinese. |
| Additional sources | Reference lists of included studies and relevant reviews were manually screened. Clinical trial registries, including ClinicalTrials.gov and the Chinese Clinical Trial Registry, were checked when feasible using combinations of "colorectal cancer", "bevacizumab", and "FOLFOX". Grey literature sources, including dissertations and conference abstracts, were screened when identifiable. | Not applicable. | Manual searching used the same core concepts: disease + bevacizumab + FOLFOX. | Dissertations, conference abstracts, reviews, and other non-eligible publication types were excluded during screening if they did not meet the predefined inclusion criteria. If the original review did not fully rerun these sources after correcting the drug term, this should be stated transparently in the manuscript or response letter. |

**Abbreviations:** MeSH, Medical Subject Headings; Emtree, Excerpta Medica thesaurus; TS, Topic; ti,ab, title/abstract.

**Important note:** If the search was originally executed with the misspelled term "Bedarzab", the authors should rerun and verify all database searches using the corrected drug name "bevacizumab" and its synonym "Avastin" to minimize the risk of missing eligible studies.
